# EEG functional connectivity for Parkinson’s disease diagnosis and non-motor symptom prediction: a machine learning approach

**DOI:** 10.3389/fnhum.2026.1921946

**Published:** 2026-09-16

**Authors:** Fanzun Meng, Shuo Liu, Xiaoyu Xiao, Chuanmin Ma, Fangchun Huang, Zhuqing Xu, Zixiang Guo, Wenjing Jiang

**Affiliations:** Department of Geriatric Medicine & Laboratory of Gerontology and Anti-Aging Research, Qilu Hospital of Shandong University, Jinan, Shandong, China

**Keywords:** EEG, functional connectivity, machine learning, non-motor symptom, Parkinson’s disease

## Abstract

**Introduction:**

Parkinson’s disease (PD) is associated with severe non-motor symptoms, including mood disorders and sleep disturbances, whose neural mechanisms are linked to dysfunction in large-scale brain networks. Functional connectivity measured by electroencephalography (EEG) can noninvasively map abnormalities at the network level.

**Methods:**

This study employed the debiased weighted phase lag index (dwPLI) to mitigate the effects of volumetric conductivity and investigated EEG functional connectivity in PD patients (*n* = 14) and healthy controls (*n* = 14) during resting and emotional states. Diagnostic performance was evaluated using L1-regularized logistic regression combined with a strict subject-level leave-one-subject-out cross-validation method.

**Results:**

The accuracy of the beta band model combining the four states reached 82.14% (*p* < 0.05, permutation test), while the accuracy of the gamma band model under the eyes-open state reached 89.29% (*p* < 0.01). Furthermore, theta band connectivity during the sadness state significantly predicted PSQI scores in PD patients.

**Discussion:**

These findings suggest that emotion-induced network dynamics may reflect sleep disturbances in PD, and establish EEG functional connectivity as a candidate neurophysiological biomarker for PD, with potential applications in both diagnostic classification and the assessment of non-motor symptoms, although validation in larger cohorts is still required.

## 1 Introduction

Parkinson’s disease (PD) is the second most common neurodegenerative disorder after Alzheimer’s disease (Poewe et al., 2017). Although the typical clinical presentation of PD involves motor symptoms (Jankovic, 2008), increasing evidence indicates that patients often exhibit various non-motor symptoms several years or even decades before these motor symptoms emerge, including emotional processing deficits and sleep disorders (Dorsey et al., 2018). PD patients exhibit significant impairments in emotional processing, particularly in recognizing and responding to negative emotions (Suzuki et al., 2006; Dara et al., 2008; Gray and Tickle-Degnen, 2010). These emotional processing deficits may correlate with structural damage in specific brain regions such as the amygdala and striatum (Tessitore et al., 2002; Dara et al., 2008; Peron et al., 2012). Furthermore, dopamine is depleted in PD, which is a key regulator of both motor function and emotional responses (Salgado-Pineda et al., 2005), closely linking the emergence of emotional disturbances.

Clinical diagnosis of PD remains largely reliant on motor symptoms, limiting sensitivity in early stages. While neuroimaging techniques like magnetic resonance imaging (MRI) and dopamine PET can detect structural and functional alterations in the brains of PD patients (Bae et al., 2021; Bidesi et al., 2021), their cost and complexity limit routine application. Electroencephalography (EEG), as an efficient, non-invasive examination method, has been widely used in studying brain mechanisms and analyzing emotions in PD patients (Bocková and Rektor, 2019; Donmez and Ozkurt, 2019; Sun et al., 2022). By applying various emotional stimuli and integrating assessment tools such as the Hamilton Depression Rating Scale (HAMD) and Hamilton Anxiety Rating Scale (HAMA), researchers can deeply explore EEG characteristics in PD subjects under emotional states and their association with emotional disorders (Yuvaraj et al., 2014a,b, 2017). Furthermore, using EEG, machine-learning approaches have reported high diagnostic accuracy (Rahman et al., 2020) and demonstrated strong performance in recognizing emotional states among PD patients (Murugappan et al., 2020).

Emotion emerges from distributed brain-circuit interactions rather than isolated regions (Moonen et al., 2017). Therefore, EEG functional connectivity, which quantifies synchrony between brain areas, may therefore provide a more fundamental measure of the emotional processing network. In healthy individuals, Wu et al. (2022) proposed novel emotion-related key subnetworks based on EEG functional connectivity analysis, achieving 95.08% emotion classification accuracy in the SEED dataset, and enabling prediction of emotional arousal and valence. Furthermore, Yuvaraj et al. (2015) study found that during negative emotional states, PD patients exhibited significantly reduced interhemispheric coherence in theta, alpha, beta, and gamma frequency bands compared to healthy controls (HC), indicating marked differences in brain functional connectivity between patients and healthy individuals during emotional states.

However, studies on brain functional connectivity in PD patients remain relatively scarce, with inconsistent results (Yuvaraj et al., 2015, 2016; Yang and Chen, 2023) and their emotion classification accuracy has yet to reach an ideal level in some reports (Yuvaraj et al., 2016), leaving substantial room for improvement. The specific objectives of this study are as follows:

1. To compare differences between PD patients and healthy controls (HCs) across different frequency bands (delta, theta, alpha, beta, gamma and all-band) and four states (eyes-closed, eyes-open, happy, and sad).
2. To evaluate whether EEG functional connectivity can reliably distinguish PD patients from HCs using rigorous subject-level cross-validation (LOSO-CV).
3. To examine the predictive value of EEG connectivity features for the severity of non-motor symptoms, including anxiety, depression, and sleep disturbances.

To address methodological challenges in small-sample EEG studies, we employed rigorous subject-level cross-validation, regularization, and multidimensional analysis across states and frequency bands.

## 2 Materials and methods

### 2.1 Participants

The overall study workflow is illustrated in Figure 1. This study enrolled 14 patients with PD and 14 healthy control participants. The diagnosis of PD was established according to the Chinese diagnostic criteria for Parkinson’s disease (2016 version). The inclusion criteria consisted of: being in the on medication period (ON period), aged between 50 and 85 years old, able to cooperate in completing the experimental process, no history of other serious neurological or psychiatric disorders, no serious cognitive deficits (MMSE scores ≥21), and normal or corrected-to-normal vision and hearing. Exclusion criteria involved severe motor disabilities or other health issues that could interfere with experiment completion, a history of epilepsy or other disorders that may cause EEG abnormalities, and use of medications other than standard PD treatments—such as drugs prescribed for psychiatric conditions or sleep disorders. All participants underwent cognitive assessment using the Mini Mental State Examination (MMSE), the Montreal Cognitive Assessment (MoCA), the Hamilton Depression Scale (HAMD), Hamilton Anxiety Scale (HAMA), and Pittsburgh Sleep Quality Index (PSQI). Statistical analyses were then performed to examine potential differences in both demographic characteristics and the scores from these clinical scales between the patient and control groups.

**Figure 1:**
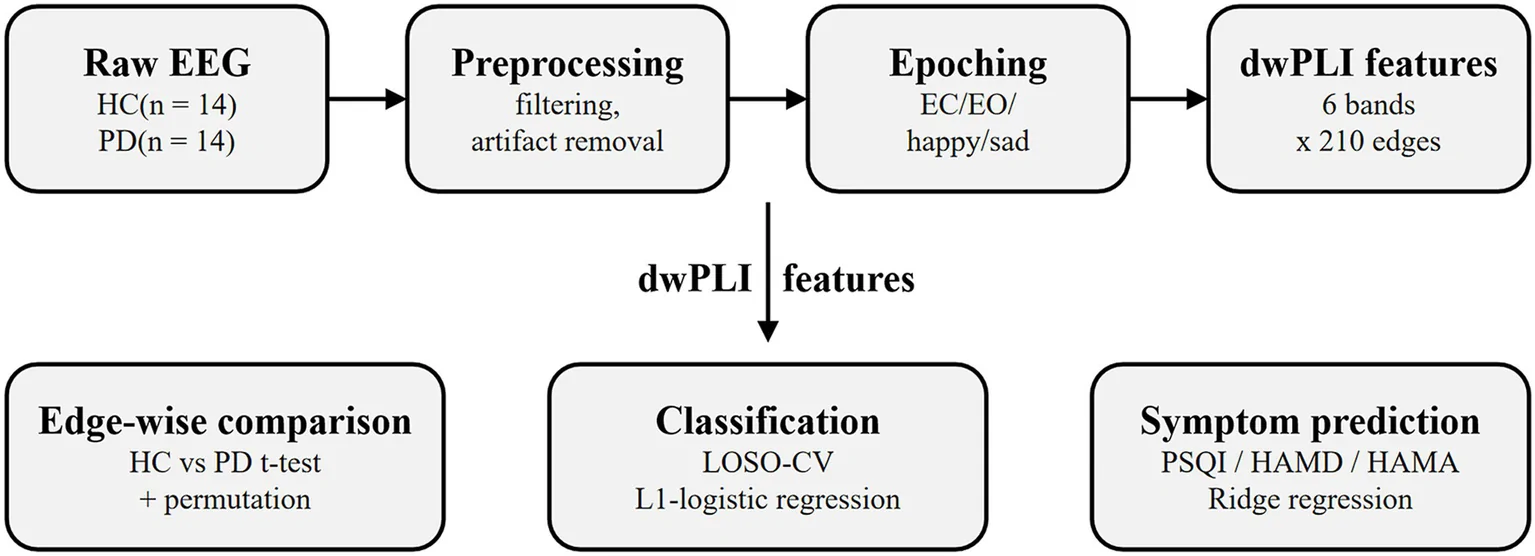
Study workflow. Raw EEG recordings from 14 HC participants and 14 participants with PD were preprocessed, categorized into four states (EC, EO, happy, sad), and dwPLI connectivity features (6 frequency bands × 210 edges) were extracted. Three downstream analyses were then conducted: edge-level between-group comparisons, diagnostic tests, and symptom prediction (PSQI/HAMD/HAMA).

### 2.2 Experimental procedure

The emotion experiment was conducted in a laboratory setting, and the EEG was recorded in four stages of subjects’ eyes closed, eyes open, happy, and sad. First, subjects underwent EEG recordings in a quiet closed-eye state for 1 min. Subsequently, EEG was recorded with eyes open for 1 min. After resting for about 5 min, subjects watched 5 min of a happy video clip and 5 min of a sad video clip.

### 2.3 EEG acquisition and preprocessing

EEG signals were recorded using a Nihon Kohden EEG-9200K system with 21 scalp electrodes placed according to the international 10–20 system, as shown in Supplementary Figure S1. The sampling rate was 500 Hz, and electrodes A1 and A2 served as the online reference. Electrode impedance was maintained below 5 kΩ throughout the recording.

EEG signals were preprocessed using the MNE toolkit for Python (Gramfort et al., 2013). Data were referenced to the earlobe electrodes (A1, A2), followed by 0.5–50 Hz bandpass filtering (FIR zero-phase filter, Hamming window) and 50 Hz notch filtering. Independent component analysis (ICA) was used to remove eye movement and muscle artifacts. Components were classified as muscle-related when the power ratio in the 30–50 Hz band exceeded the mean plus two standard deviations of the power ratios across all components. Continuous data were segmented into 2-s epochs corresponding to four conditions: eyes-closed, eyes-open, happy, and sad. The first 10 s of the happy and sad conditions were discarded to minimize adaptation effects. Epochs with peak-to-peak amplitudes exceeding 300 μV or below 1 μV were automatically rejected.

### 2.4 Brain functional connectivity network

Functional connectivity was quantified using the debiased weighted phase lag index (dwPLI) (Vinck et al., 2011), with calculations covering six frequency bands: delta (1–4 Hz), theta (4–8 Hz), alpha (8–13 Hz), beta (13–30 Hz), gamma (30–50 Hz), and all-band (1–50 Hz). The dwPLI connectivity matrices were computed for each epoch and then averaged across epochs within each participant and state, yielding a 21 × 21 connectivity matrix for each subject under each state. The lower triangular portion of the matrix (210 connections) was extracted as the feature vector for subsequent analyses.

### 2.5 Feature construction and diagnosis

For each participant and each state, the connectivity matrices were averaged across epochs to generate a single feature vector (210 dimensions). For the comprehensive analysis of the four states, the feature vectors from the four states were concatenated for each participant to form a composite feature vector (840 dimensions).

Diagnosis was performed using L1-regularized logistic regression. Model performance was evaluated using leave-one-subject-out cross-validation (LOSO-CV): in each fold, data from one participant were used as the test set, while data from the remaining 27 subjects were used as the training set. The model was fitted on the training set and applied to the test set.

### 2.6 Prediction of non-motor symptom severity

Ridge regression was used to predict HAMA, HAMD, and PSQI scores based on EEG connectivity features. For each participant, an averaged feature vector was paired with the corresponding clinical score. The model was evaluated using the same LOSO-CV framework.

### 2.7 Statistical methods

Statistical significance of the diagnostic accuracy was assessed using a permutation test (1,000 iterations), which was conducted by randomly shuffling group labels and repeating the full LOSO-CV process. The *p*-value was defined as the proportion of permutations that yielded an accuracy rate ≥ the true accuracy rate. To avoid overly conservative corrections across all analysis combinations, permutation tests were performed only on the best-performing combination in each analysis. Bootstrap resampling (1,000 iterations) was used to estimate the 95% confidence interval for the best-performing result.

For regression models, the same permutation procedure (1,000 iterations) was used to evaluate the *R*^2^ value, and the 95% confidence interval was estimated using bootstrap resampling.

For edge-wise group comparisons, independent-sample *t*-tests (Welch’s *t*-test) were performed for each state with FDR correction (*q* < 0.05).

### 2.8 Computational complexity

The model used in this study has a relatively low computational burden. The training time complexity of L1-regularized logistic regression (solved using coordinate descent) is of the order of O(*n*·*p*·*k*), where *n* is the number of training samples, *p* is the feature dimension (210 for single-state analysis and 840 for four-state analysis), and *k* is the number of iterations; prediction requires only a single linear projection, with a time complexity of O(p). The time complexity of ridge regression is comparable. Experiments show that on a personal computer equipped with an Intel Core i7-12700 processor and 64 GB of memory, the training time for the four-state classifier (840 features) is approximately 1 millisecond per LOSO-CV fold, and the entire 28-fold cross-validation is completed in less than 0.03 s; the inference time for a single new participant is less than 0.1 milliseconds. These results indicate that the proposed method is suitable for near-real-time clinical applications.

### 2.9 Ethical statement

This study was registered at ClinicalTrials.gov (ID: NCT06295263; Release Date: May 6, 2025) and received ethical approval from the Ethics Committee of Shandong University Qilu Hospital (Approval No.: KYLL-202203 (XZ)-028-2). All participants were fully informed of the experimental procedures and provided written informed consent prior to the commencement of the study.

## 3 Results

### 3.1 Participant characteristics

Table 1 summarizes the demographic and clinical characteristics of the participants. Statistical comparisons revealed no significant differences between the HC and PD groups in terms of age (*p* = 0.683), MMSE scores (*p* = 0.132), MoCA scores (*p* = 0.289), HAMD scores (*p* = 0.113), and PSQI scores (*p* = 0.919) were not significantly different. In contrast, scores on the HAMA were significantly higher the PD group compared to the control group (*p* = 0.005). After automatically removing outliers, the HC group retained 4,843 epochs and excluded 295; the PD group retained 4,081 epochs and excluded 753.

**Table 1:** Demographic and clinical profiles of PD patients and HC participants.

Characteristic	PD	HC	*p*-value
Sample size, *N*	14	14	NA
Female/male	7/7	7/7	NA
Age, yr	65.14 ± 6.72	66.14 ± 6.08	0.683
MMSE	25.36 ± 2.95	27.00 ± 2.63	0.132
MoCA	20.86 ± 5.11	22.86 ± 4.66	0.289
HAMD	8.43 ± 3.82	6.00 ± 4.02	0.113
HAMA	12.00 ± 5.94	5.93 ± 4.41	0.005*
PSQI	8.36 ± 5.40	8.57 ± 5.67	0.919
Hoehn & Yahr stage	2.07 ± 0.27	NA	NA
UPDRS-part III	34.43 ± 10.32	NA	NA
Duration of disease, yr	3.93 ± 2.83	NA	NA

Data are expressed as mean ± standard deviation. PD, Parkinson’s disease; HC, healthy control; MMSE, Mini-Mental State Examination; MoCA, Montreal Cognitive Assessment; HAMD, Hamilton Depression Scale; HAMA, Hamilton Anxiety Scale; PSQI, Pittsburgh Sleep Quality Index; UPDRS, Unified Parkinson’s Disease Rating Scale; *p < 0.05 means significant difference existed.

### 3.2 EEG functional connectivity characteristics

Edge-wise group comparisons were performed using independent-sample *t*-tests across all frequency bands and states. After FDR correction (*q* < 0.05), no single edge passed the multiple comparison correction in any frequency band or state. In the uncorrected analyses (*p* < 0.05), the alpha band exhibited the highest number of nominally significant margins across all conditions, followed by the beta and delta bands. Most nominally significant connections (67.2%) indicated that the PD group exhibited stronger connectivity than the HC group (PD > HC). This hyperconnectivity pattern was most pronounced in the temporo-frontal, temporo-parietal, and occipital-central pathways across theta, beta, and gamma bands, particularly during eyes-open and emotional states. PD < HC patterns were less common and primarily occurred in the alpha and gamma bands during the eyes-closed resting state, involving fronto-central and temporo-occipital connections. Figure 2 shows the spatial distribution of these nominally significant edges across frequency bands.

**Figure 2:**
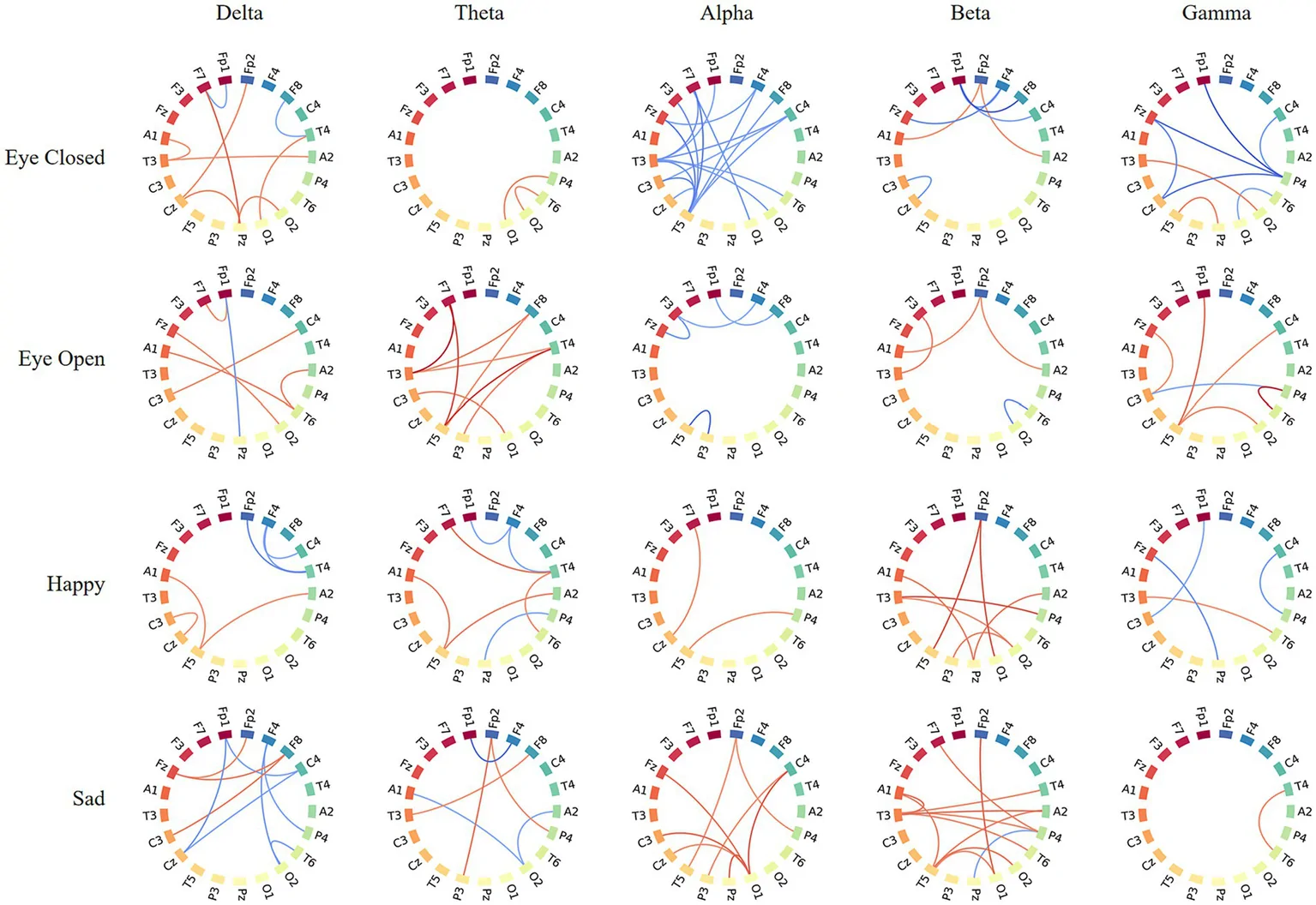
Comparison of the brain functional connectivity between PD patients and HC across four states. In each subplot, red lines represent connections exhibiting significantly stronger connectivity in PD compared to HC, while blue lines indicate connections that are weaker in PD than in HC (*p* < 0.05). The deeper the color of the lines, the greater the difference in strength of the PD compared to the HC. Each abbreviation label corresponds to an EEG electrode location in the international 10–20 system. The letters denote scalp regions: Fp, frontal pole; F, frontal; C, central; T, temporal; P, parietal; O, occipital. Odd numbers denote the left hemisphere, even numbers denote the right hemisphere, and “z” denotes the midline electrode.

### 3.3 Diagnostic performance

Diagnostic performance was evaluated across six frequency bands and four states using L1-regularized logistic regression combined with LOSO-CV. In the single-state analysis, as shown in Table 2, the gamma band in the eyes-open state achieved the highest accuracy of 89.29% (*p* = 0.002, 95% CI: [78.57, 100.00%]). Other notable combinations included beta during happy (82.14%) and theta during sad (71.43%). Accuracy rates in the delta and alpha bands were generally lower across most states (35.71–50.00%).

**Table 2:** Single-state diagnostic accuracy (%) across frequency bands and states.

Band	Eyes-closed	Eyes-open	Happy	Sad
Delta	39.29	35.71	46.43	39.29
Theta	53.57	67.86	60.71	71.43
Alpha	46.43	39.29	50.00	35.71
Beta	53.57	35.71	82.14	53.57
Gamma	50.00	89.29	57.14	42.86
All	39.29	50.00	50.00	64.29

As shown in Table 3, in the comprehensive analysis of the four states, the beta band had the highest accuracy of 82.14% (*p* = 0.029, 95% CI: [67.86, 96.43%]), outperforming the theta band (60.71%), gamma band (60.71%), alpha band (42.86%), all-band (42.86%), and delta band (32.14%).

**Table 3:** Combined four-state diagnostic performance across frequency bands.

Band	Accuracy (%)	Sensitivity (%)	Specificity (%)
Delta	32.14	28.57	35.71
Theta	60.71	57.14	64.29
Alpha	42.86	35.71	50.00
Beta	82.14	85.71	78.57
Gamma	60.71	64.29	57.14
All	42.86	35.71	50.00

### 3.4 Prediction of non-motor symptom severity

LOSO-CV-based ridge regression was employed to predict HAMA, HAMD, and PSQI scores using EEG connectivity features. For the best-performing combinations in each group, permutation tests (1,000 iterations) and bootstrap resampling (1,000 iterations) were conducted, respectively. In the PD group, PSQI was significantly predicted by three combinations: sad + all-band (*R*^2^ = 0.595, 95% CI: [0.09, 0.79], *p* < 0.001), happy + theta band (*R*^2^ = 0.557, 95% CI: [0.29, 0.73], *p* < 0.001), and sad + theta band (*R*^2^ = 0.497, 95% CI: [−0.09, 0.67], *p* = 0.003). In the HC group, two combinations reached significance: eyes-closed + theta for HAMA (*R*^2^ = 0.402, 95% CI: [−0.46, 0.70], *p* = 0.009) and happy + alpha for PSQI (*R*^2^ = 0.271, 95% CI: [−0.38, 0.52], *p* = 0.019). The best-performing combinations in the PD and HC groups are summarized in Table 4 and presented in Figure 3. Complete regression results including all combinations are provided in Supplementary Tables S1, S2.

**Table 4:** Significant regression results for non-motor symptom prediction.

Scale	Group	State + band	*R* ^2^	95% CI	*p*-value
PSQI	PD	Sad + all	0.595	[0.09, 0.79]	<0.001
PSQI	PD	Happy + theta	0.557	[0.29, 0.73]	<0.001
PSQI	PD	Sad + theta	0.497	[−0.09, 0.67]	0.003
HAMA	HC	Eyes-closed + theta	0.402	[−0.46, 0.70]	0.009
PSQI	HC	Happy + alpha	0.271	[−0.38, 0.52]	0.019

CI, confidence interval (bootstrap, 1,000 iterations). HAMD, Hamilton Depression Scale; HAMA, Hamilton Anxiety Scale; PSQI, Pittsburgh Sleep Quality Index.

**Figure 3:**
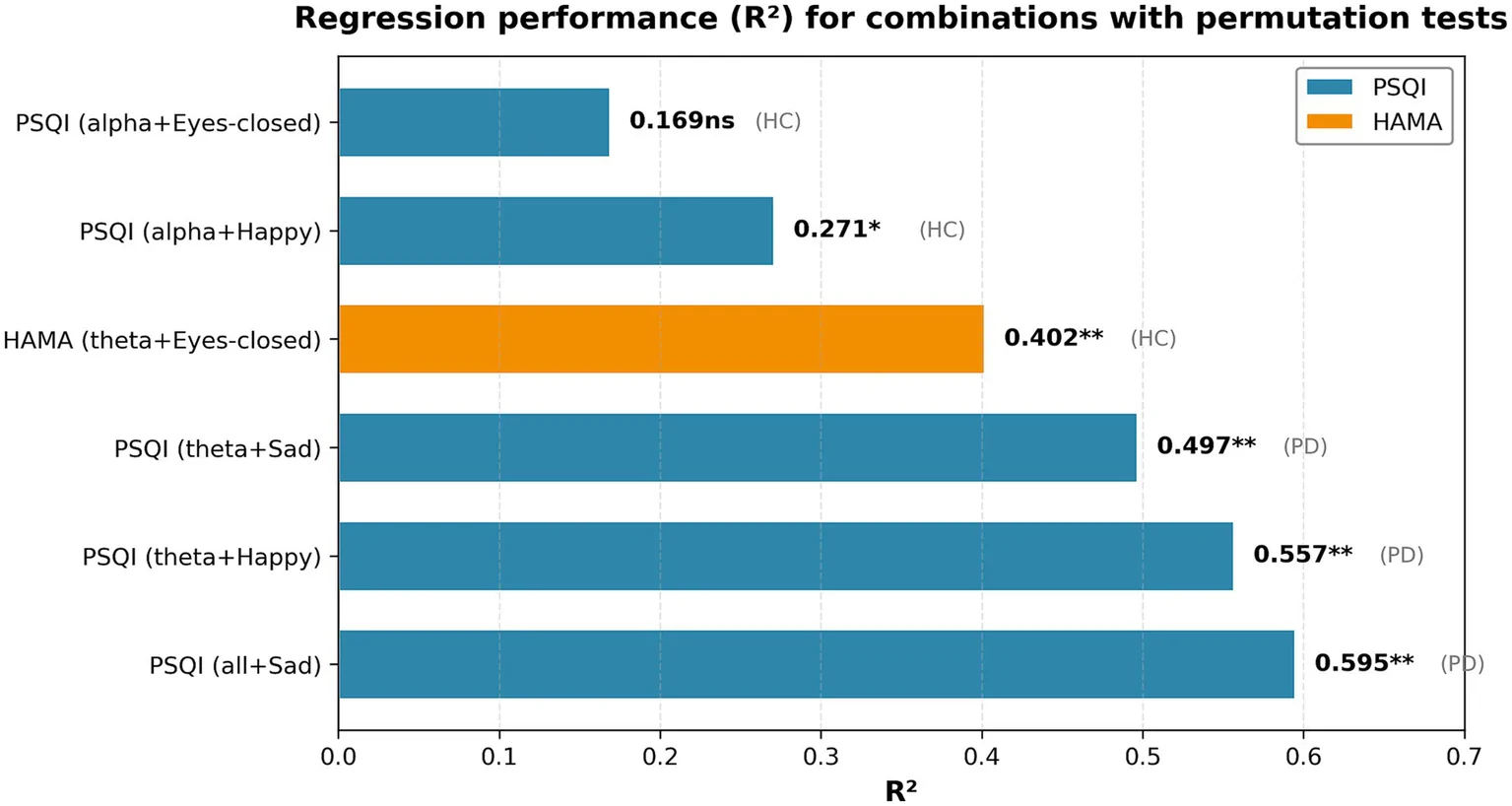
Regression performance (*R*^2^) for combinations that underwent permutation testing. Only combinations with permutation tests are shown (Top 3 in each group: PD left; HC right). Bars represent *R*^2^ values for each frequency-state combination; color indicates the clinical scale (PSQI or HAMA). Significance levels from permutation tests (1,000 iterations) are shown: **p < 0.01, *p < 0.05, ns, not significant.

## 4 Discussion

This study utilized dwPLI technology to investigate EEG changes in functional connectivity of PD patients and evaluated the technical’s application value in diagnosis and the prediction of non-motor symptoms through a rigorous subject-level cross-validation framework. In terms of research methodology, this study has made several advances over previous research: we adopted a rigorous LOSO-CV framework to prevent data leakage, used dwPLI to mitigate volume conduction effects, and employed a multi-state design to capture frequency- and state-dependent connection patterns. The results indicate that EEG functional connectivity, particularly in the beta and gamma bands, can reliably distinguish PD patients from healthy controls, with the highest single-state accuracy of 89.29% (gamma band, eyes-open) and a robust combined four-state accuracy of 82.14% (beta band). Furthermore, all-band connectivity during the sad state demonstrated significant predictive power for PSQI scores (*R*^2^ = 0.595), suggesting that emotion-induced EEG network dynamics may capture clinically relevant information about sleep disturbances in PD.

Edge-wise group comparisons revealed frequency- and state-dependent connectivity changes in PD. Overall, 67.2% of nominally significant edges showed higher connectivity in PD compared to HC, indicating a general trend toward network hyperconnectivity across multiple frequency bands and states (Waninger et al., 2020). This hyperconnectivity pattern was most pronounced in the temporo-frontal, temporo-parietal, and occipital-central pathways, particularly in the theta, beta, and gamma bands during eyes-open and emotional states. In contrast, reduced connectivity in the PD group (PD < HC) was less common and occurred primarily in the alpha and gamma bands during the eyes-closed state, involving fronto-central and temporo-occipital connections. These directional differences suggest that PD-related connectivity changes cannot be simply characterized as global hyper- or hypoconnectivity, but rather as a state- and frequency-dependent reorganization of large-scale networks (Conti et al., 2026). Differences in EEG connectivity changes across studies have also been documented (Yuvaraj et al., 2015; Iyer et al., 2020), likely attributable to methodological variations, patient heterogeneity, or differences in analytical metrics. Our analyses were based on dwPLI method, which effectively suppresses zero-latency connectivity caused by volume conduction (Vinck et al., 2011). The distributed nature of these changes—involving multiple edges rather than isolated connections—is consistent with the view that PD involves network-level dysfunction affecting multiple large-scale systems (Conti et al., 2023).

Consistent with previous reports of enhanced resting-state beta and gamma synchrony in PD patients (Waninger et al., 2020), the diagnostic analyses further indicated that the beta and gamma bands carried the most discriminative information. The gamma band during eyes-open achieved the highest single-state accuracy (89.29%), while the beta band in the combined four-state model achieved a robust accuracy of 82.14%. The superior performance of the gamma band during the eyes-open state is consistent with the known role of gamma oscillations in sensory and cognitive processing and may reflect PD-related alterations in visual cortical networks during the eyes-open resting state (Tropini et al., 2011). The beta-band result, derived from integrating information across all four experimental conditions, provides a more comprehensive characterization of PD-related network alterations (Conti et al., 2026). Together, these findings suggest that EEG functional connectivity—particularly in the beta and gamma bands—contains reliable discriminative information for PD, and that integrating multiple brain states can enhance the stability of diagnostic features (Qiu et al., 2022).

Table 5 compares recent functional connectivity studies on PD classification based on EEG. Although our dwPLI method used only 21 scalp electrodes, it still achieved competitive performance (89.29%), whereas most previous studies employed a 64-channel high-density EEG setup (Conti et al., 2023; Tülay, 2024; Zhao et al., 2024; Conti et al., 2026). Notably, we employed a multi-state design that combined resting-state and emotional-state conditions, which differs from the resting-state paradigm predominantly used in previous studies. Furthermore, compared to the k-fold cross-validation strategy commonly used in the literature, the leave-one-subject-out cross-validation method adopted in this study provides a more rigorous assessment of generalization to new individuals.

**Table 5:** Comparison of EEG-based functional connectivity studies for PD classification.

Study	Connectivity metric	Frequency bands	PD/HC (*N*)	Channels	Classifier	CV method	Best accuracy (%)
Conti et al. (2023)	wPLI	*θ*, *α*, *β*, low-*γ*	26/20	64	NBS-predict (LDA)	LOOCV	89
Tülay (2024)	Coherence (CRC)	*δ*, *θ*, *α*, *β*, *γ*	21/20	64 (30 used)	Ensemble (RF, AdaBoost, GB)	10-fold stratified	80.5
Zhao et al. (2024)	PCC + PLV	Broadband (4–12 Hz)	52/48	64	GSP-GCNs (Deep Learning)	5-fold	90.2
Alves et al. (2025)	Coherence, Pearson, Spearman, CCA, Ledoit–Wolf	*δ*–*γ* (Broadband)	15/16	32	MLP, CNN	10-fold stratified + 75/25 split	99
Conti et al. (2026)	wPLI	*θ*, *α*, *β*, low-*γ*, high-*γ*	140/57	64	SVM	Nested LOOCV + external validation	99
Current study	dwPLI	*δ*, *θ*, *α*, *β*, *γ* (All-band)	14/14	21	L1-regularized logistic regression	LOSO-CV	89.29

Common emotion disorders in PD patients, including anxiety, depression, and sleep disorders, have been shown to alter brain functional connectivity networks (Chen et al., 2019; Carey et al., 2021; Liu et al., 2022). For example, studies have shown that anxiety is associated with structural and functional changes in both the hypothesized fear and the limbic cortico-striato-thalamocortical circuits (Carey et al., 2021), and neuroimaging suggests that changes in functional connectivity in the ventral striatal subregion of PD patients may serve as a marker for depression (Wang et al., 2022). In addition, functional connectivity in the anterior cingulate cortex and right straight gyrus is reduced in patients with PD with sleep disorders (Chen et al., 2019). In the regression analysis, it was confirmed that theta band connectivity is the strongest predictor of PSQI scores in patients with PD aside from the all-band analysis. This finding is consistent with evidence that theta oscillations play a fundamental role in sleep processing (Kim and Park, 2025), with theta activity specifically altered in PD patients (Memon et al., 2023; Zhang et al., 2024).

However, the predictive performance for HAMD and HAMA was relatively limited, with optimal *R*^2^ values of 0.284 and 0.049, respectively. This likely reflects the multidimensional and multidomain nature of emotional symptoms in PD, which involves complex interactions among motor, autonomic, cognitive, and psychosocial factors that cannot be fully captured by scalp-level EEG connectivity alone (Carey et al., 2021; Ahmad et al., 2023). Notably, the predictive ability of the healthy control group was negligible across all scales, confirming that the specificity of the association between EEG and symptoms is limited to the PD population. These results suggest that while EEG connectivity during emotional challenge shows potential for tracking sleep quality in PD, prediction of emotional symptoms likely requires additional clinical covariates or larger samples, and should be interpreted as exploratory rather than confirmatory.

Several methodological limitations are worth considering. First, although the dwPLI technique mitigates volumetric conduction effects, our findings are based on scalp-level connectivity and do not involve source reconstruction. Therefore, all interpretations are limited to the topological distribution of electrodes at the scalp level. Second, the small sample size limits the generalizability of the classification and regression results. Due to the limited sample size (*n* = 14 per group), we did not perform analyses adjusted for covariates (e.g., HAMA, MoCA, age, or sex), which may affect the interpretation of differences between groups. Third, all PD patients were assessed while on medication, which may have influenced functional connectivity patterns. Fourth, this study did not include a disease-specific control group; therefore, the specificity of the observed connectivity patterns for PD could not be assessed. Despite these limitations, rigorous methodological controls—including subject-level cross-validation, permutation tests, and bootstrap confidence intervals—ensure the reliability of the reported results, which can be interpreted as exploratory evidence.

Finally, evidence suggests that beta band activity may serve as a marker of emotional and behavioral control and may predict responsiveness to neuromodulatory treatments in mental disorders. Importantly, subdividing the beta range into distinct subbands can reveal functionally different connectivity patterns and treatment responses that may be obscured when beta activity is analyzed as a single broad frequency band. This frequency-specific perspective may also be relevant for the treatment of PD, as it has been shown that deep brain stimulation targeting specific beta-frequency ranges can be more effective than conventional high-frequency stimulation in PD (Habelt et al., 2025; Habelt, 2026). Future studies are therefore encouraged to conduct such frequency-resolved analyses on larger, more diverse study cohorts to further optimize EEG-based biomarkers providing more refined diagnostic and prognostic information for PD and the assessment of non-motor symptoms.

## 5 Conclusion

This study demonstrates that EEG functional connectivity, particularly in the beta and gamma bands, can reliably distinguish PD patients from healthy controls, with the highest accuracy in a single state being 89.29% (gamma band, eyes-open state) and a robust accuracy of 82.14% across the four combined states (beta band). Theta band connectivity during the sadness state predicted PSQI scores, suggesting that emotion-induced dynamic changes in EEG may reflect sleep quality in PD patients. These findings support the use of EEG functional connectivity as a potential neurophysiological biomarker for the assessment of PD, although validation in larger sample sizes is still required.

## Data Availability

The EEG dataset supporting the findings of this study is publicly available in Zenodo at https://doi.org/10.5281/zenodo.21998315. The code implementing the results of this work is openly available on GitHub at https://github.com/FanzunMeng/EEG-FC-PD-Diagnosis-and-Symptom-Prediction.git.
