# Supplementary material for: EEG functional connectivity for Parkinson’s disease diagnosis and non-motor symptom prediction: a machine learning approach
Source: Front Hum Neurosci. 2026 Sep 16;20:1921946. doi: 10.3389/fnhum.2026.1921946 (PMC13624067; doi:10.3389/fnhum.2026.1921946)
Supplement: Supplementary file 1 [file Data_Sheet_1.DOCX]

Supplementary Material

Figure S1. Distribution of 21 EEG electrodes on the scalp according to the International 10-20 System.

Table S1. Complete regression results for all frequency-state-scale combinations in PD group.

| Band | State | Scale | R² | RMSE | MAE |
| --- | --- | --- | --- | --- | --- |
| delta | eyes-closed | PSQI | -0.4716 | 6.3135 | 5.1398 |
| delta | eyes-closed | HAMD | -0.5259 | 4.5439 | 3.5311 |
| delta | eyes-closed | HAMA | -1.0740 | 8.2371 | 6.2697 |
| delta | eyes-open | PSQI | -0.1195 | 5.5067 | 5.0342 |
| delta | eyes-open | HAMD | -0.3576 | 4.2859 | 3.5159 |
| delta | eyes-open | HAMA | -0.9369 | 7.9603 | 6.7962 |
| delta | happy | PSQI | -0.3926 | 6.1416 | 4.9603 |
| delta | happy | HAMD | 0.0285 | 3.6257 | 3.0666 |
| delta | happy | HAMA | -0.3605 | 6.6714 | 5.5407 |
| delta | sad | PSQI | 0.2465 | 4.5176 | 3.9486 |
| delta | sad | HAMD | -0.8499 | 5.0031 | 4.2602 |
| delta | sad | HAMA | -0.4842 | 6.9681 | 5.8635 |
| theta | eyes-closed | PSQI | -0.3345 | 6.0122 | 5.2896 |
| theta | eyes-closed | HAMD | -0.3787 | 4.3191 | 3.8255 |
| theta | eyes-closed | HAMA | -0.8916 | 7.8665 | 7.1072 |
| theta | eyes-open | PSQI | -0.3897 | 6.1354 | 4.8974 |
| theta | eyes-open | HAMD | -0.5390 | 4.5633 | 3.4916 |
| theta | eyes-open | HAMA | -0.4041 | 6.7774 | 5.6305 |
| theta | happy | PSQI | 0.5573 | 3.4627 | 2.8784 |
| theta | happy | HAMD | 0.0752 | 3.5374 | 2.9713 |
| theta | happy | HAMA | -0.5211 | 7.0543 | 6.0063 |
| theta | sad | PSQI | 0.4966 | 3.6928 | 3.2767 |
| theta | sad | HAMD | -0.0398 | 3.7508 | 3.3575 |
| theta | sad | HAMA | -0.5188 | 7.0490 | 5.5832 |
| alpha | eyes-closed | PSQI | -1.2692 | 7.8399 | 7.0095 |
| alpha | eyes-closed | HAMD | -1.0559 | 5.2742 | 4.1893 |
| alpha | eyes-closed | HAMA | -0.7261 | 7.5145 | 6.0554 |
| alpha | eyes-open | PSQI | -0.4348 | 6.2341 | 5.2265 |
| alpha | eyes-open | HAMD | -0.5864 | 4.6331 | 3.7039 |
| alpha | eyes-open | HAMA | -0.1210 | 6.0558 | 5.0087 |
| alpha | happy | PSQI | -0.4689 | 6.3078 | 5.2019 |
| alpha | happy | HAMD | -0.7651 | 4.8870 | 3.9403 |
| alpha | happy | HAMA | -0.2374 | 6.3624 | 5.4143 |
| alpha | sad | PSQI | -0.1978 | 5.6961 | 4.5805 |
| alpha | sad | HAMD | -0.5501 | 4.5797 | 3.8068 |
| alpha | sad | HAMA | -0.8827 | 7.8480 | 6.4252 |
| beta | eyes-closed | PSQI | 0.2024 | 4.6480 | 4.2889 |
| beta | eyes-closed | HAMD | 0.1127 | 3.4649 | 3.1818 |
| beta | eyes-closed | HAMA | -0.2482 | 6.3902 | 4.9740 |
| beta | eyes-open | PSQI | -0.5801 | 6.5421 | 5.4664 |
| beta | eyes-open | HAMD | -0.1624 | 3.9659 | 3.3933 |
| beta | eyes-open | HAMA | -0.2995 | 6.5202 | 5.2151 |
| beta | happy | PSQI | 0.1026 | 4.9302 | 4.1358 |
| beta | happy | HAMD | 0.1374 | 3.4163 | 3.0852 |
| beta | happy | HAMA | -0.9582 | 8.0039 | 6.8324 |
| beta | sad | PSQI | -1.2922 | 7.8796 | 6.4525 |
| beta | sad | HAMD | -0.0998 | 3.8576 | 2.9929 |
| beta | sad | HAMA | -0.3921 | 6.7485 | 5.0198 |
| gamma | eyes-closed | PSQI | -0.3287 | 5.9992 | 5.4114 |
| gamma | eyes-closed | HAMD | -0.3960 | 4.3461 | 3.7182 |
| gamma | eyes-closed | HAMA | 0.2395 | 4.9880 | 4.4090 |
| gamma | eyes-open | PSQI | -0.4916 | 6.3563 | 5.5515 |
| gamma | eyes-open | HAMD | -0.8883 | 5.0547 | 4.6326 |
| gamma | eyes-open | HAMA | -0.6228 | 7.2862 | 6.3466 |
| gamma | happy | PSQI | -0.9651 | 7.2958 | 6.3657 |
| gamma | happy | HAMD | -0.3275 | 4.2382 | 3.3607 |
| gamma | happy | HAMA | -1.2455 | 8.5709 | 6.8616 |
| gamma | sad | PSQI | -0.5344 | 6.4469 | 5.1369 |
| gamma | sad | HAMD | 0.4164 | 2.8100 | 2.4923 |
| gamma | sad | HAMA | -1.1213 | 8.3304 | 5.7811 |
| all | eyes-closed | PSQI | -0.5408 | 6.4602 | 5.3685 |
| all | eyes-closed | HAMD | -0.5562 | 4.5888 | 4.1014 |
| all | eyes-closed | HAMA | -0.2298 | 6.3428 | 5.3122 |
| all | eyes-open | PSQI | -0.0804 | 5.4096 | 4.8540 |
| all | eyes-open | HAMD | -0.0293 | 3.7318 | 3.1002 |
| all | eyes-open | HAMA | -0.5603 | 7.1446 | 5.6912 |
| all | happy | PSQI | 0.3730 | 4.1211 | 3.0610 |
| all | happy | HAMD | -0.2899 | 4.1778 | 3.3635 |
| all | happy | HAMA | -0.5631 | 7.1509 | 5.7009 |
| all | sad | PSQI | 0.5954 | 3.3105 | 3.0243 |
| all | sad | HAMD | 0.2837 | 3.1132 | 2.6601 |
| all | sad | HAMA | 0.0490 | 5.5777 | 4.1321 |

Table S2. Complete regression results for all frequency-state-scale combinations in HC group.

| Band | State | Scale | R² | RMSE | MAE |
| --- | --- | --- | --- | --- | --- |
| delta | eyes-closed | PSQI | -0.8922 | 7.5112 | 6.9102 |
| delta | eyes-closed | HAMD | -1.2175 | 5.7674 | 4.7760 |
| delta | eyes-closed | HAMA | -0.9473 | 5.9314 | 5.2728 |
| delta | eyes-open | PSQI | -0.4679 | 6.6157 | 5.3028 |
| delta | eyes-open | HAMD | -0.3143 | 4.4401 | 3.4452 |
| delta | eyes-open | HAMA | -0.7973 | 5.6982 | 4.4714 |
| delta | happy | PSQI | -1.0352 | 7.7899 | 7.0285 |
| delta | happy | HAMD | -0.7956 | 5.1898 | 4.5013 |
| delta | happy | HAMA | -1.1533 | 6.2371 | 5.4309 |
| delta | sad | PSQI | -1.4927 | 8.6210 | 6.7100 |
| delta | sad | HAMD | -2.1454 | 6.8688 | 5.7303 |
| delta | sad | HAMA | -1.6762 | 6.9534 | 5.4103 |
| theta | eyes-closed | PSQI | -0.4920 | 6.6697 | 5.5855 |
| theta | eyes-closed | HAMD | -0.2652 | 4.3563 | 3.6545 |
| theta | eyes-closed | HAMA | 0.4015 | 3.2882 | 2.8708 |
| theta | eyes-open | PSQI | -0.2594 | 6.1280 | 5.1307 |
| theta | eyes-open | HAMD | -0.9389 | 5.3929 | 4.0990 |
| theta | eyes-open | HAMA | -0.7749 | 5.6626 | 3.9943 |
| theta | happy | PSQI | -1.6829 | 8.9439 | 7.5189 |
| theta | happy | HAMD | -2.2554 | 6.9880 | 6.1714 |
| theta | happy | HAMA | -1.6867 | 6.9670 | 6.1394 |
| theta | sad | PSQI | -0.3044 | 6.2365 | 5.5368 |
| theta | sad | HAMD | -1.7996 | 6.4803 | 5.8559 |
| theta | sad | HAMA | -1.0426 | 6.0748 | 5.3200 |
| alpha | eyes-closed | PSQI | 0.1685 | 4.9791 | 4.0492 |
| alpha | eyes-closed | HAMD | -0.7482 | 5.1208 | 4.2467 |
| alpha | eyes-closed | HAMA | -0.5530 | 5.2968 | 4.3519 |
| alpha | eyes-open | PSQI | -1.8292 | 9.1846 | 7.4743 |
| alpha | eyes-open | HAMD | -4.0495 | 8.7030 | 5.6370 |
| alpha | eyes-open | HAMA | -2.3601 | 7.7913 | 5.3228 |
| alpha | happy | PSQI | 0.2709 | 4.6625 | 3.8743 |
| alpha | happy | HAMD | -0.2865 | 4.3929 | 3.5270 |
| alpha | happy | HAMA | -0.7603 | 5.6393 | 5.1211 |
| alpha | sad | PSQI | -0.6703 | 7.0571 | 5.9294 |
| alpha | sad | HAMD | -0.5960 | 4.8928 | 4.1133 |
| alpha | sad | HAMA | -0.2553 | 4.7622 | 4.1300 |
| beta | eyes-closed | PSQI | -1.4021 | 8.4629 | 7.1833 |
| beta | eyes-closed | HAMD | -0.8765 | 5.3054 | 4.1120 |
| beta | eyes-closed | HAMA | -1.4166 | 6.6075 | 5.9612 |
| beta | eyes-open | PSQI | -0.4261 | 6.5209 | 5.6681 |
| beta | eyes-open | HAMD | -0.7434 | 5.1138 | 4.3155 |
| beta | eyes-open | HAMA | -0.1560 | 4.5699 | 3.4337 |
| beta | happy | PSQI | -2.3334 | 9.9695 | 7.3249 |
| beta | happy | HAMD | -2.5804 | 7.3284 | 5.5942 |
| beta | happy | HAMA | -3.1089 | 8.6158 | 7.5116 |
| beta | sad | PSQI | -1.4523 | 8.5510 | 6.4112 |
| beta | sad | HAMD | -1.4527 | 6.0655 | 4.2311 |
| beta | sad | HAMA | -1.5644 | 6.8066 | 5.6368 |
| gamma | eyes-closed | PSQI | -0.1061 | 5.7428 | 4.8278 |
| gamma | eyes-closed | HAMD | -0.1756 | 4.1993 | 3.5358 |
| gamma | eyes-closed | HAMA | -0.2613 | 4.7736 | 3.6994 |
| gamma | eyes-open | PSQI | -1.7477 | 9.0512 | 7.5249 |
| gamma | eyes-open | HAMD | -2.3958 | 7.1371 | 5.0377 |
| gamma | eyes-open | HAMA | -0.4602 | 5.1363 | 3.9996 |
| gamma | happy | PSQI | -0.7971 | 7.3200 | 6.4705 |
| gamma | happy | HAMD | -0.7167 | 5.0744 | 4.2864 |
| gamma | happy | HAMA | -1.0229 | 6.0454 | 4.8057 |
| gamma | sad | PSQI | -2.1058 | 9.6231 | 7.6868 |
| gamma | sad | HAMD | -4.4857 | 9.0712 | 6.5043 |
| gamma | sad | HAMA | -2.7261 | 8.2047 | 5.8605 |
| all | eyes-closed | PSQI | -0.2445 | 6.0915 | 5.4511 |
| all | eyes-closed | HAMD | -0.3446 | 4.4909 | 3.5658 |
| all | eyes-closed | HAMA | -0.4486 | 5.1157 | 4.6728 |
| all | eyes-open | PSQI | -1.9187 | 9.3287 | 7.7275 |
| all | eyes-open | HAMD | -1.1100 | 5.6259 | 4.7287 |
| all | eyes-open | HAMA | -0.2845 | 4.8173 | 3.8501 |
| all | happy | PSQI | -0.2224 | 6.0371 | 4.7703 |
| all | happy | HAMD | -0.5080 | 4.7561 | 3.7476 |
| all | happy | HAMA | -0.4516 | 5.1210 | 4.5111 |
| all | sad | PSQI | -0.2843 | 6.1882 | 5.1766 |
| all | sad | HAMD | -0.7054 | 5.0577 | 4.1882 |
| all | sad | HAMA | -0.3720 | 4.9787 | 4.6187 |
